# Organic–Inorganic Magnetic Nanoparticles Based on Magnetite Coated with Molecularly Imprinted Polymers for Drug Delivery Systems

**DOI:** 10.1021/acsomega.4c09515

**Published:** 2025-05-19

**Authors:** Sandra Ramírez-Rave, Leticia Antonio Gutiérrez, Iván D. Rojas-Montoya, Ma Josefa Bernad-Bernad, Jesús Gracia-Mora

**Affiliations:** † Departamento de Química Inorgánica, Facultad de Química, 7180 UNAM, Avenida Universidad 3000, 04510 Ciudad de México, México; ‡ Departamento de Farmacia, Facultad de Química, UNAM, Avenida Universidad 3000, 04510 Ciudad de México, México

## Abstract

New magnetic organic/inorganic hybrid nanoparticles were prepared by coating the magnetite surface with molecularly imprinted polymers of different monomers (methacrylic acid, itaconic acid, 1-vinylimidazole, 4-vinylpyridine). Magnetite was synthesized by coprecipitation, and polymeric coatings were induced by thermal heating and magnetic hyperthermia. The resultant nanocomposites were tested as Drug Delivery Systems (DDS) of 6-mercaptopurine (6-MP), displaying an interesting behavior depending on (a) the synthesis method used for their preparation and (b) the conditions employed for the release test. DDS based on methacrylic acid displayed the best performance in the release of 6-MP in all cases, but the rationalization of a magnetite MIP (molecularly imprinted polymer) system of this monomer enhanced the release yield by approximately 50% compared with the nonimprinted system (NIP) prepared with the same monomer. New MNP obtained are able to behave as DDS under magnetic hyperthermia with a moderate performance in contrast to the release in the absence of magnetic field.

## Graphical abstract

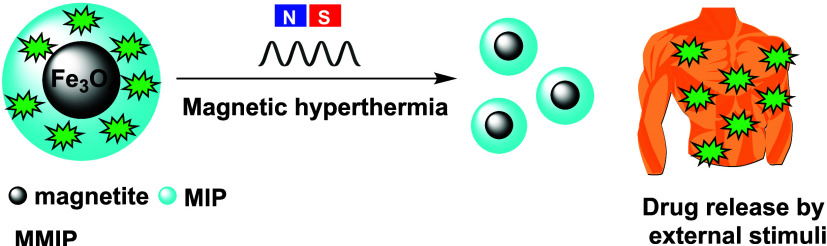

## 1 Introduction

MIPs are polymeric species that mimic biological receptors for the recognition of a specific substrate.^1^ There has been a boom in the use of these materials in recent years due to their versatility and applications in different fields, especially analytical chemistry. Nevertheless, directed studies on the design of new DDS employing this rationalization have a promising future.^2^ The main advantage of MIPs is the rationalization of cavities formed in the polymeric network, which mimic exactly the template molecule in size, form, and functional groups because the resulting binding sites are specific for recognition of the substrate.^3^ However, MIP lacks vectorization or directionality features; therefore, junctions must be built between MIP and other materials that exhibit these characteristics.

In this direction, magnetic nanoparticles (MNP) based on superparamagnetic iron materials (like magnetite, Fe_3_O_4_) are playing a prevalent role in nanomedicine advancements because of their biocompatibility, indomitable low-cost production, versatile chemistry, and physicochemical stability, which make them unique for use in magnetic resonance imaging (MRI) and magnetic hyperthermia sources or drug delivery systems.^4,5^ In addition, the superparamagnetic properties in a certain range of MNP sizes can be controlled and directed to desired pathological regions using external magnetic fields. Enhancing biomedical features and loading drug capacity is possible by coating MNPs with organic polymers, which can also be synthesized as MIPs. An attractive advantage of magnetic molecularly imprinted particles (MMIPs) is that the magnetic properties of MNPs can be combined with the ability of molecular recognition of MIPs in a single hybrid functional structure; which results in a DDS able to provide the drug in a targeting site, diminishing the dose and improving treatment efficacy with a decrease in the side effects of normal tissues.^6^

In recent years, the design and synthesis of diverse magnetic materials for DDS, including chemotherapy agents, have been explored and tested,^7^ but the development of MMIPs for this purpose is emerging.

Some efforts have been published in this direction^8^ employing different anticancer drugs as templates for the development of MMIPs. Dramou et al.^9^ synthesized MMIPs using epirubicin as a template for its application as a DDS. Although they did not employ an external magnetic field for either the fabrication of the DDS or the release of the drug, their study demonstrated notable performance in controlling the release of epirubicin over time in the resultant systems. This work highlights the versatility of MMIPs in drug delivery applications, even in the absence of magnetic stimuli, showcasing their potential for sustained and controlled therapeutic delivery. Griffete and co-workers^10^ reported the synthesis of a MMIP based on magnetite and acrylamide for DDS, using doxorubicin as a template anticancer agent. This innovative material demonstrated active control over drug release through the application of magnetic hyperthermia. By combining the controlled drug release capabilities of nonthermosensitive MIPs with the magnetic properties of iron oxide, the system enables precise regulation of doxorubicin release. Hashemi-Moghaddam et al.^11^ synthesized MMIPs by coating magnetic nanoparticles with polydopamine for the controlled delivery of 5-Fluorouracil (5-FU) in a mouse breast cancer model. The results demonstrated that the application of an external magnetic field significantly enhanced the localized release of 5-FU from the magnetic nanoparticles, improving its therapeutic efficacy in the treatment of murine breast adenocarcinoma. Hassanpour and co-workers^12^ developed a magnetic molecularly imprinted polymer (MMIP) for the controlled release of azidothymidine (AZT) under the influence of an alternating magnetic field. This material demonstrated excellent *in vitro* performance against cancer cell lines, highlighting its potential as an effective therapeutic delivery system. Kaamyabi et al.^13^ produced a pH- and thermosensitive MMIP, [poly­(NIPAAM@Fe_3_O_4_ MNPs/TMSPMC/DOX)], designed as a drug carrier for doxorubicin (DOX) delivery. While this material demonstrated precise behavior as DDS, the study did not report the use of an alternating magnetic field in its application. Other relevant contributions to this topic have been reported in the literature before with different anticancer and antineoplastic drugs.^14–18^

6-Mercaptopurine (6-MP) is an antineoplastic drug that belongs to the antimetabolites group; this molecule presents immunosuppressant properties and is employed for leukemia treatment. It is a class II drug (low solubility and high permeability) and presents a half-life of 1.5 h. This compound has potent acid–base properties (p*K*_a_: 2.99 and 9.5) and offers binding sites for coordinating metals.^19^ A few studies have reported the use of this drug in MMIPs for analytical purposes^20^ but not for DDS.

This study presents the design and synthesis of novel MMIPs using 6-MP as a template, evaluating their efficacy as DDS. Two fabrication strategies for magnetic nanoparticles (MNPs) were investigated: (1) a conventional reflux-based polymerization, which promoted coating on magnetite nanoparticles synthesized previously by coprecipitation, and (2) a magnetic hyperthermia-assisted polymerization, where heat generated under an alternating magnetic field facilitated the coating process. Nonimprinted polymers served as controls. The MMIPs exhibited efficient magnetic separation, enabling the purification of the resulting materials. Furthermore, MMIPs synthesized with methacrylic acid (MMIP-MA) and 4-vinylpyridine (MMIP-4VP) demonstrated promising DDS performance under alternating magnetic fields.

## 2 Materials and Methods

Iron dichloride tetrahydrate (FeCl_2_·4H_2_O, >99%), iron chloride hexahydrate (FeCl_3_·6H_2_O, ≥99%), ethylene glycol dimethyl acrylate (EGDMA, 98%), methacrylic acid (99%), itaconic acid (≥99%), 1-vinylimidazole (≥99%), 4-vinylpyridine (95%), and 6-mercaptopurine hydrochloride (6-MP, 98%) were obtained from Sigma-Aldrich. Azobis­(isobutyronitrile) (AIBN) was provided by AKZO NOBEL Chemicals. S.A. de C.V.

Acetonitrile and ethanol were acquired from J.T. Baker. Methacrylic acid, 4-vinylpyridine monomers, and EGDMA were passed through an inhibitor removal resin (Alfa Aesar).

### 2.1 Synthesis of Magnetite Nanoparticles

The synthesis of magnetite particles was carried out using the coprecipitation method, 0.50 g of FeCl_2_·4H_2_O and 1.296 g of FeCl_3_·6H_2_O (2:1 stoichiometric ratio) were weighed and dissolved in 24.5 mL water. The mixture was placed in an N_2_ atmosphere and heated to 85 °C with vigorous stirring. Finally, approximately 10 mL of a 2 M NaOH solution was added to precipitate the magnetite. After the reaction was completed, the particles were washed with water until the pH reached approximately 7, and then separated magnetically using a 14,000 G neodymium magnet. The synthesized particles were dried at 40 °C for 1 h in a thermostatic vacuum desiccator (Vacuo-Temp, J.P. SELECTA) and then for 24 h at room temperature.

### 2.2 Synthesis of MMIP and NIP Materials

Polymeric coatings on magnetite surface were synthesized employing 200 mg of the magnetite particles that were resuspended in 100 mL of acetonitrile, this solution was sonicated for 10 min, and 0.2 mmol of functional monomer [approximately 20 μL of 4-vinylpyridine (VP), 1-vinylimidazole (VIN), and methacrylic acid (MA), and 30 mg of itaconic acid (ITA)] was added. Followed by the addition of 21.5 mg of 6-mercaptopurine (0.125 mmol) dissolved in a mixture of methanol MeOH/acetonitrile (3:7).

The mixture was heated at 45 °C and 250 rpm for 3 h. After this time, the particles were precipitated by the action of a magnet, and the reaction residues were washed three times with acetonitrile. Resulting MMIPs were labeled in the text based on the monomer used for their synthesis, with the prefix MMIP (MMIP-VP, MMIP-VIN, MMIP-MA, and MMIP-ITA).

The stoichiometric ratio between the drug, functional monomer and cross-linker is 1:4:20, this ratio is widely used in most studies of imprinted polymers, and in our own studies, it has been seen that it is efficient for an adequate imprinting and cross-linking process.

NIPs were prepared employing the same methodology, except for the part of the addition of 6-MP, and were labeled on the text depending on the monomer employed for the synthesis with the prefix MNIP (MNIP-VP, MNIP-VIN, MNIP-MA, and MNIP-ITA).

### 2.3 Synthesis of a Hybrid System via Hyperthermia of Magnetite Nanoparticles

The polymeric coatings on the nanoparticles were done using the hyperthermia-generating capacity of magnetite, exposing the nanoparticles to an alternating magnetic field during the synthesis in a zero-voltage switching (ZVS) Mazilli oscillator circuit (alternating magnetic field generator) connected to a 40 V output power supply with 15 W of power. This equipment was previously used and was characterized by our research group. The experiment was conducted in a thermostated flask surrounded by a coil made of 5/16 in. copper tubing (*N* = 5). The frequency and generated magnetic field were measured using an InfiniiVision 2000 X-series oscilloscope (Agilent) equipped with an MC162 EMC EMI RFI probe. To carry out the experiment, 50 mg of nanoparticles were weighed and sonicated in 40 mL of acetonitrile for 10 min. The dispersed particles were then placed in a thermostatized flask, and 0.1 mmol of methacrylic acid (10 μL) was added to functionalize them. The equipment was put into operation while the particles were stirred at 250 rpm on an orbital shaker (Dragon Scientific, DSR-10) to prevent their precipitation. Functionalization was performed in 2 h. 0.1 mmol more of methacrylic acid, 0.78 mmol of EGDMA (110 μL), and 10 mg of AIBN were added to the mixture and stirred well. The system was then placed under a nitrogen atmosphere and placed back into an alternative magnetic field generator equipment. Samples were collected at 0 (functionalized nanoparticles, FUN-HPT), 3 (NIP-HPT-3H), and 24 h (NIP-HPT-24H) to verify whether polymerization had occurred. Control particles (without hyperthermia, in the same conditions) were also synthesized and taken at 1 (CNT-1H) and 24 h (CNT-24H) for suitable study.

### 2.4 Characterization

Fourier transform infrared (FTIR) spectra of each compound were obtained using a PerkinElmer Spectrum 400 FTIR/FIR instrument from 4000 to 400 cm^–1^. Elemental analysis was performed using a PerkinElmer 2400 Elemental Analyzer for CHNS using cystine as the calibration standard. Both methods were applied to the powder samples.

Differential scanning calorimetry (DSC) to determine the thermal properties of the synthesized materials was employed, materials were tested under a heating cycle from 30 to 300 °C at a rate of 20 °C/min in a DSC1 Mettler Toledo instrument.

Morphological analysis of the particles was performed using a scanning electron microscope (SEM) from JEOL, model JSM-6010LA operated at 10 kV and 15 mA with a working distance of 10 mm. The samples were analyzed dry and were previously coated with a thin layer of gold to prevent static charge. The particle size distribution and ζ-potential were measured using a particle size and ζ-potential analyzer (Zetasizer Nano ZS, Malvern Instruments) equipped with a He–Ne laser at a wavelength of 633 nm and a temperature of 25 °C. The samples were prepared in ultrapure water and analyzed in triplicate.

Transmission electron microscopy (TEM) experiments were performed in a JEOL JEM-ARM200F transmission electron microscope operated at 200 keV. The images were analyzed using Gatan DigitalMicrograph software version 3.30.2016.0 and ImageJ software.

Thermogravimetric analysis was performed using a TA Q50 instrument at a temperature interval from 25 to 800 °C at a rate of 10 °C/min under a N_2_ atmosphere. Weight loss was measured as a function of temperature.

Energy-dispersive X-ray absorption spectroscopy (XAS) was used to analyze the structure of the nanoparticle systems, and measurements were performed at the XAS beamline at the Elettra synchrotron light source in Trieste (Italy). The samples were prepared as pressed pellets and analyzed in total fluorescence mode.

The crystal structure of magnetite was characterized using a D8 ADVANCE X-ray diffractometer (BRUKER AXS) in θ–θ mode. A Cu Kα radiation source with λ = 1.54 Å was used. Phase identification was performed using MATCH! software.

The particle size was determined by dynamic light scattering (DLS) in a Zetasizer Nano ZS using glass and dip cells for measurements. The nanoparticles were dispersed in deionized water (approximately 0.2 mg of nanoparticles/mL) and sonicated for 10 min, and the measurements were performed at 25 °C.

### 2.5 Release Studies of the Systems

To evaluate synthesized materials as drug delivery systems, *in vitro* release studies were conducted on them. 70 mg of each system (MIPs without 6-MP and NIPs) were separately placed in 50 mL of a 6-MP solution in MeCN/MeOH medium (3:7) with a concentration of 2 × 10^–3^ M under stirring at 250 rpm and 25 °C for 12 h. After treatment, particles were separated magnetically and washed with 20 mL of MeOH. Finally, the systems were dried at room temperature for 24 h.

After loading each system with the drug, respective release studies were performed. For this purpose, 10 mg of each drug-loaded system was separately placed in contact with 40 mL of phosphate buffer at pH 7.4 with a constant temperature of 37 °C (PolyScience temperature controller) to simulate physiological conditions. Stirring was carried out at 250 rpm in an orbital shaker. The release kinetics of each system were evaluated in triplicate, with samples collected at various time points (0.25, 0.5, 1, 2, 3, 4, 5, 6, 7, 24, and 30 h). The amount of 6-MP released was determined using a calibration curve constructed with UV–vis spectroscopy across different concentrations.

### 2.6 Release Studies Using Magnetic Hyperthermia

To evaluate the effect of magnetic hyperthermia on drug release, *in vitro* drug release studies were conducted using MMIP systems. Ten milligrams of each system were separately placed in a thermostatic vessel containing 40 mL of phosphate buffer at pH 7.4. The vessel was placed in the center of the coil in an alternating magnetic field generator, maintaining the temperature at 37 °C and agitation at 250 rpm to evaluate only the effect of hyperthermia on the release of 6-MP, exposing the nanoparticles to an alternating magnetic field of 107 kHz and 17.7 G during the test. The released 6-MP was quantified as described in Section 2.6.

## 3 Results and Discussion

### 3.1 Synthesis and Characterization of Magnetic Hybrid Nanoparticles

The synthesis of magnetic MIPs was carried out first by modifying the magnetite surface with different functional monomers. The subsequent steps are illustrated in Figure 1.

**Figure 1:**
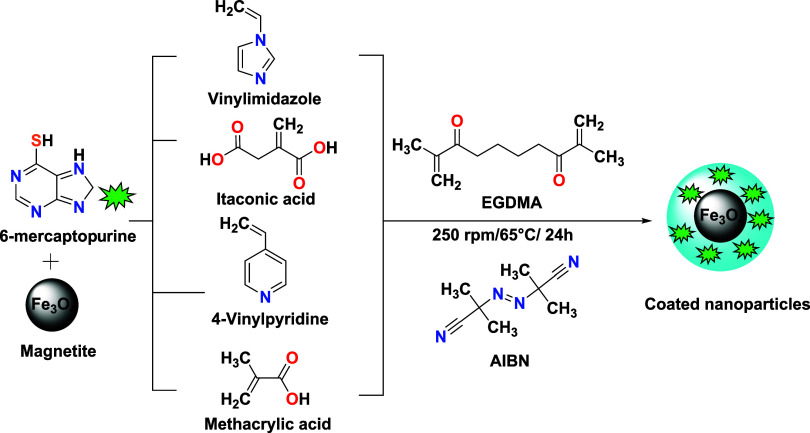
Scheme of the synthesis of magnetic systems coated with molecularly imprinted polymers (6-MP).

For the synthesis of nonimprinted systems, the addition of 6-MP to the mixture is simply omitted. Particles synthesized in the presence of 6-MPs (MMIPs) were washed to remove the drug before characterization and subsequent adsorption studies. Once the particles were coated with the polymer and the template molecule (6-MP), the latter was extracted from the MMIPs, and the systems were characterized to verify whether the coating was successfully performed and to study the new features of the obtained hybrid material. The results obtained for testing the coated particles using different methods are described below. To determine whether the magnetite nanoparticles had a polymer coating, FTIR analysis was performed on the eight systems, and the results are shown in Figure 2.

**Figure 2:**
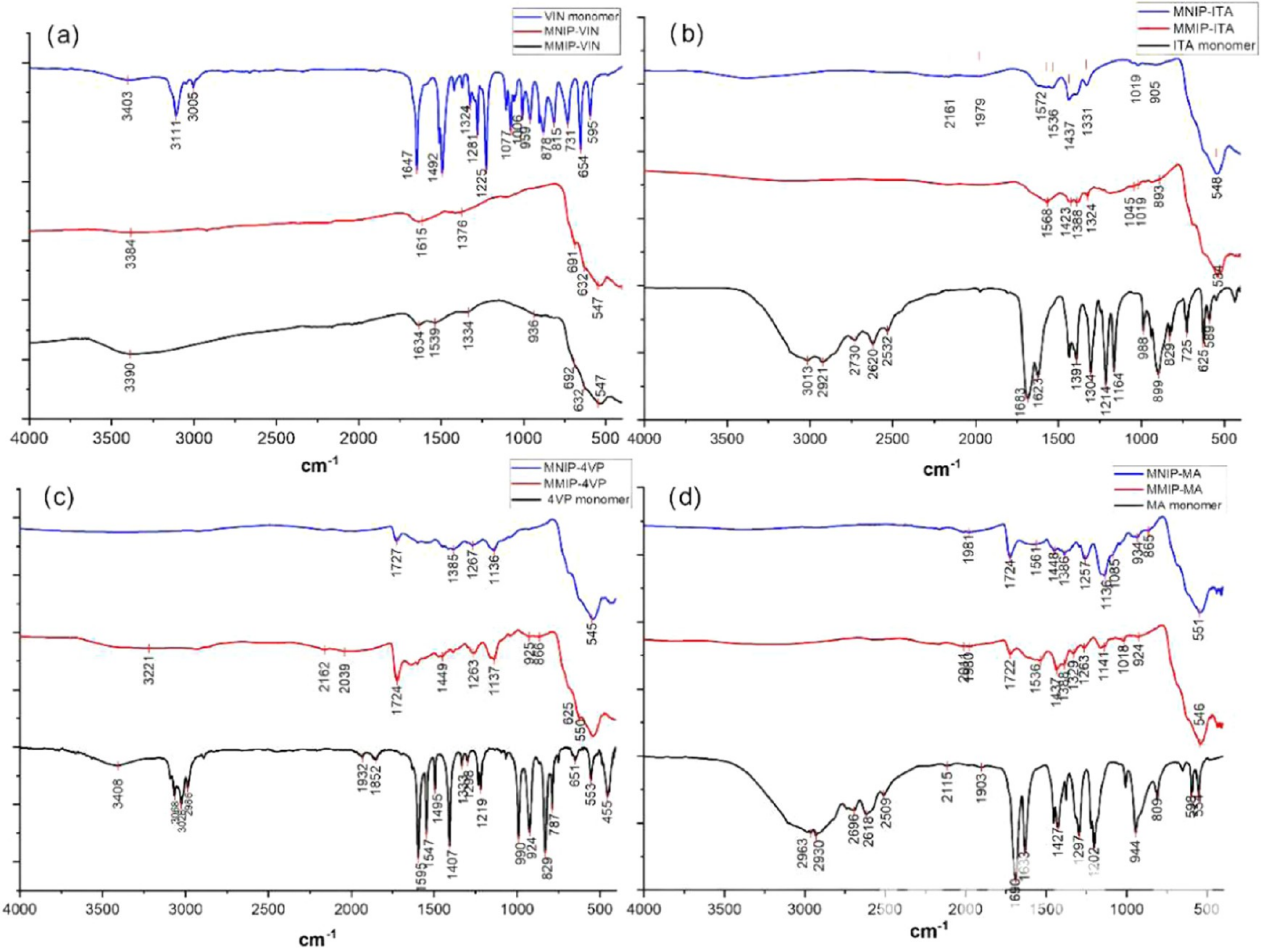
(a) FTIR spectra of the MMIP-VIN (black) and MNIP-VIN systems and of the functional monomer vinylimidazole (blue). (b) FTIR spectra of the MMIP-4VP and MNIP-4VP (blue) systems and the functional monomer 4-vinylpyridine (black). (c) FTIR spectra of the MMIP-ITA and MNIP-ITA (blue) systems and the functional monomer itaconic acid (black). (d) FTIR spectra of the MMIP-MA (blue) and MNIP-MA systems and of the functional monomer methacrylic acid (black).

In Figure 2a, signals at 3384 cm^–1^ (MNIP-VIN) and 3390 cm^–1^ (MMIP-VIN) are attributed to the presence of the functional monomer vinylimidazole, as this region shows stretches associated with aromatic amines through the asymmetric stretching of the −C=NH group, and bands at 1615 and 1634 cm^–1^ confirm the previous assignment, as bands in these regions are associated with C=N stretching. The signals at 1334 and 1376 cm^–1^ are associated with the out-of-plane stretching of the methyl groups (−CH_3_) present in the polymer. Finally, the disappearance of the bands at 3005 and 3111 cm^–1^ in the spectra of the coated particles was expected since these bands correspond to the –HC=CH_2_ stretching of the functional monomer structure, which is susceptible to radical reactions. It should be mentioned that the intensity of the bands was very low compared to the characteristic band at 547 cm^–1^, which was attributed to magnetite and associated with the Fe–O stretching. This low signal intensity was due to the significantly different proportions of polymer-magnetite, indicating that the coating on the nanoparticle was much lower in mass than the nanoparticle, as expected. Lipert et al. reported metallic complexes with polyvinylimidazole and did comparable assignations to their compounds. On the other hand, Nan and collaborators also described this type of signals for poly­(vinylimidazole) grafted on magnetic nanoparticles.^21,22^

The infrared (IR) spectra of the MMIP-4VP and MNIP-4VP systems (Figure 2b), low-intensity signals are observed, which can be attributed to the small amount of polymer coating on the particles. The absorption bands at 1724 cm^–1^ (MMIP-4VP) and 1723 cm^–1^ (MNIP-4VP) correspond to the –C=O stretching of the carbonyl group, and the signals at 1137 and 1263 cm^–1^ in MMIP-4VP and at 1136 and 1267 cm^–1^ for MNIP-4VP correspond to the symmetric and asymmetric stretching of the −C–O– linkage of the ester groups in EGDMA.^23^ Therefore, these signals indicate the cross-linker in the polymer coating on the nanoparticles. Finally, bands at 545 and 553 cm^–1^ indicate the presence of magnetite particles, as they are representative of the stretching of the Fe–O bond, as mentioned earlier.

In Figure 2c, for the hybrid systems in which itaconic acid was used as the functional monomer, signals at 534 cm^–1^ (MMIP-ITA) and 548 cm^–1^ (MNIP-ITA) were observed, which were attributed to the Fe–O stretching of magnetite. On the other hand, signals at 1331 and 1437 cm^–1^ for MNIP-ITA and at 1324 and 1423 cm^–1^ for MMIP-ITA are attributed to the presence of the carboxylic acid group and are associated with the torsion/stretching of the C=O group, respectively.^24^ Due to the low signal intensity, it was not possible to assign additional bands to confirm the presence of the polymer on the nanoparticles.

Figure 2d shows the IR spectra of MMIP-MA and MNIP-MA systems. Signals at 1722 and 1724 cm^–1^ are observed corresponding to the –C=O stretching of the carbonyl group, as well as bands at 1141 and 1263 ^25^ and 1136 and 1257 cm^–1^ (blue), which correspond to the symmetric and asymmetric stretching of the −C–O– group of EGDMA.^26^ Similar to the previously described spectra, the presence of magnetite nanoparticles was confirmed by the signals at 546 and 551 cm^–1^.

XRD analysis was performed on the systems to identify any changes in the crystalline structure of the magnetite particles after coating with different polymers. Characterization and phase identification of the XRD patterns were performed using MATCH! software. Referring to the JPDS 00–019–0629, a 90% correlation with magnetite was observed. The obtained patterns for the synthesized magnetite and systems are shown in Figures 3 and 4.

**Figure 3:**
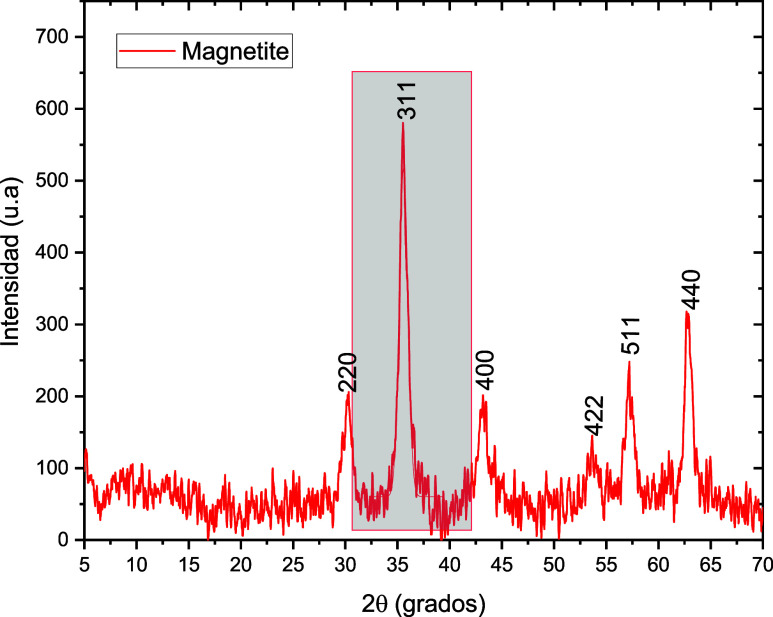
XRD patterns of synthesized magnetite.

**Figure 4:**
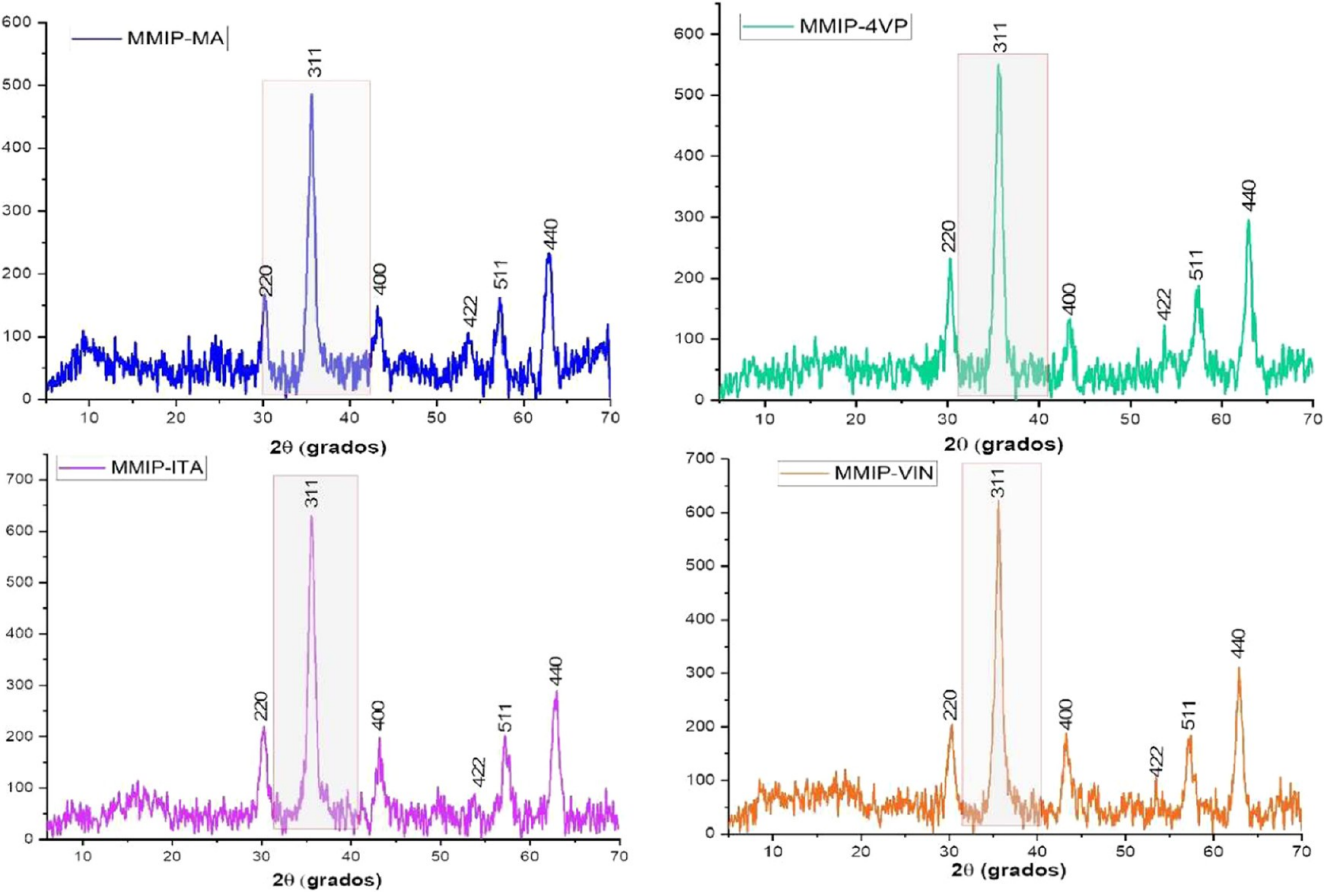
XRD patterns of the MMIP-MA, MMIP-4VP, MMIP-MA, and MMIP-MA systems. In the top right corner of each image, a Gaussian fit to the peak of maximum intensity is shown (shaded area).

The Miller indices for the diffraction patterns (220), (311), (400), (422), (511), and (440) show the characteristic crystal planes of magnetite with a cubic spinel structure in all the systems.^27^ The XRD patterns were also used to calculate the average crystallite size of the nanoparticles by employing the Debye–Scherrer equation with a Gaussian fit to the maximum intensity peak for each diffraction pattern (311 plane). Crystallite sizes of the systems are given in Table 1.

**Table 1:** Average Crystallite Sizes of the Synthesized Systems Obtained Using the Debye–Scherrer Equation

system	2θ (deg)	FWHM	crystallite size (nm)
magnetite	35.57	0.9257	9.42
MMIP-4VP	35.62	0.9657	9.03
MMIP-MA	35.56	0.92672	9.41
MMIP-VIN	35.61	0.94547	9.22
MMIP-ITA	35.59	0.8897	9.8

The particle size of the synthesized systems was determined by DLS, and the ζ-potential was determined to evaluate their dispersion stability. The results are shown in Table 2.

**Table 2:** Particle Size and ζ-Potential of the Systems Using the DLS Technique

system	average size (nm)	PDI	ζ-potential (mV)
MMIP-MA	193.5	0.396	–18
MNIP-MA	145.9	0.363	–9.44
MMIP-ITA	136.6	0.358	–11.7
MNIP-ITA	229.9	0.410	–17.5
MMIP-1VIN	141.5	0.353	–15.7
MNIP-1VIN	125.5	0.265	–26.8
MMIP-4VP	147.3	0.341	–16
MNIP-4VP	184.8	0.444	–16.4

As shown in Table 2, both the MMIPs and MNIPs coated generally exhibited particle sizes in the range of 130–230 nm. These values are considerably larger than the sizes of the magnetite particles, which are around 10 nm in size, suggesting that agglomerates formed as a technique can measure the hydrodynamic radius of the particles.^28^ The polydispersity Index (PDI) for all systems was less than 0.5, indicating that the analyzed samples were monodisperse, indicating uniform population size. Lastly, nanoparticles with a ζ-potential between −10 and +10 mV are considered nearly neutral, whereas those with ζ-potentials greater than +30 mV and less than −30 mV are considered strongly cationic and anionic, respectively.^29^ Systems with values between −30 and +30 mV tend to form agglomerates because of weaker electrostatic repulsion between them. Negative ζ-potential values were obtained in all cases but were never lower than −30 mV, consistent with the rapid agglomeration of the system.

To better visualize the synthesized systems and obtain more accurate particle size estimates, samples were studied by TEM and SEM. The analysis of the obtained micrographs was performed using the Gatan DigitalMicrograph 3.01.598.0 software. Clusters of around 50 nm were observed. Additionally, a *Z* contrast analysis confirmed the presence of the polymer, as the technique is based on the difference in the molecular weights of the polymers. The micrographs obtained for all systems are shown below (Figure 5).

**Figure 5:**
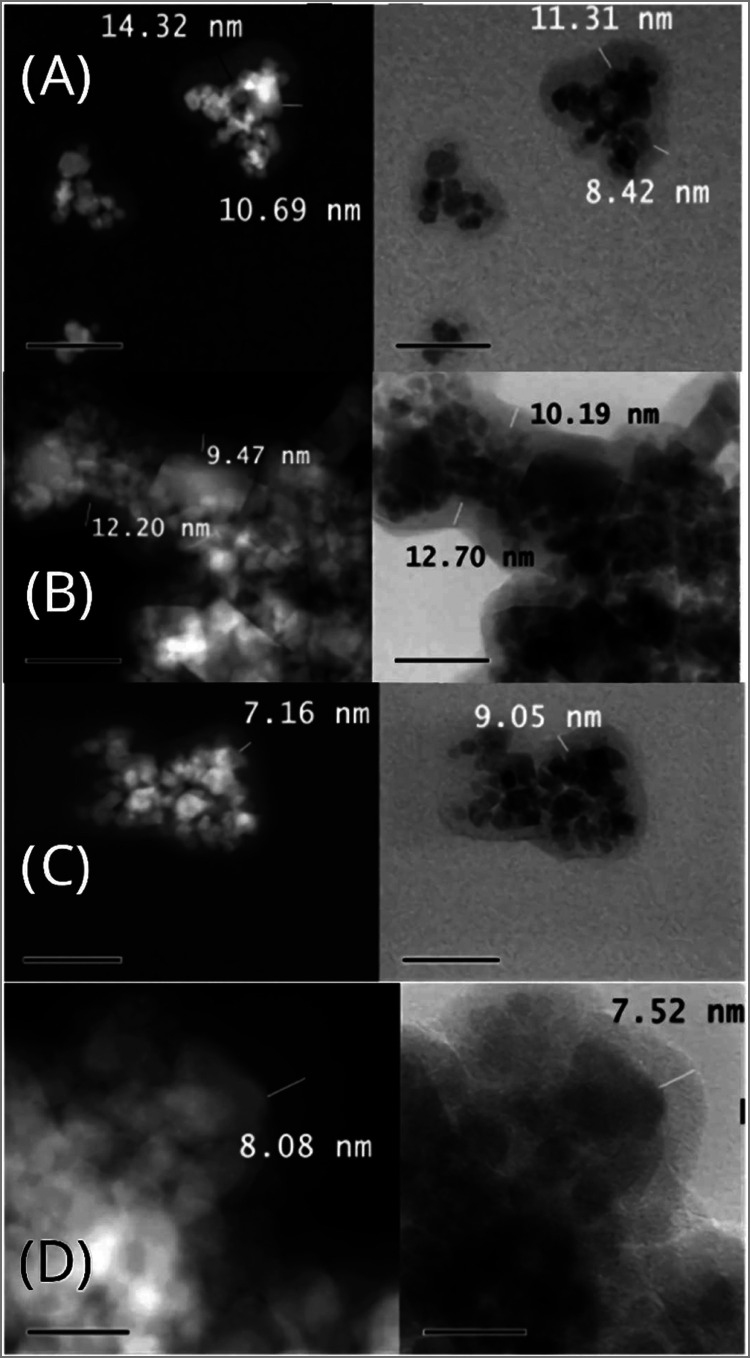
(a) TEM micrographs of MMIP-MA, (b) MMIP-4VP, (c) MMIP-1VIN and (d) MMIP-ITA. In all cases, images are displayed in the dark (left) and light (right) fields.

Figure 5 shows the TEM images of the resulting MMIPs. In general, nanocomposites formed by agglomerated polymer-coated nanoparticles are observed. The *Z*-contrast method in dark and light fields allows the differentiation of the phases present in the material. For all systems, in the left part of the images shown in Figure 5, the material phase with a higher atomic weight displays brighter and, on the right, the opposite behavior is observed (lower atomic weight is brighter). This is because Fe has a higher atomic weight than C (predominant element in polymer) and this difference in mass is presumed to be responsible for the diffuse halo exhibited in the polymer particles. The widths of the polymeric coatings were not uniform for all cases, observing a rage of 8–10 nm, but in the case of the MMIP-MA material, the highest value of 14.32 nm was noticed.

In Figure 6 MMIP-MA SEM micrograph is shown, similar images were obtained for each MIP system, where agglomerated structures of irregular shapes were observed due to the polymer presence, for which it was not possible to perform a structural analysis of particles or a dispersion size particle estimation using this method.

**Figure 6:**
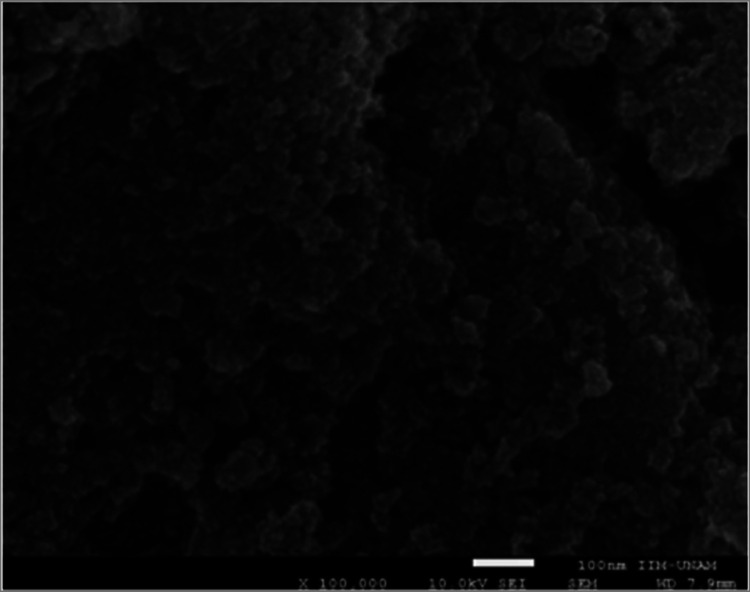
SEM micrograph of MMIP-MA system.

Energy dispersive X-ray spectroscopy (EDS) analysis was performed to analyze the amount of polymer coating on the nanoparticles. The results obtained for the MMIP-MA system are presented in Figure 6. The elements in an MMIP-MA sample are displayed in this figure. EDS was performed to identify elements that demonstrate the presence of nanoparticles and coatings, resulting in images of iron, carbon, and oxygen. This demonstrates that the coating observed in the STEM images is polymer.

The amount of iron in the sample was sufficient for observation by this technique. However, despite the clear polymer layer shown in the *Z*-contrast of the carbon and oxygen mappings (c) and (d), respectively, Figure 7 indicates background noise and does not clearly define the nanoparticles. This was because the mass of the polymer was proportionally smaller than that of the iron oxide, and thus the resolution was not satisfactory.

**Figure 7:**
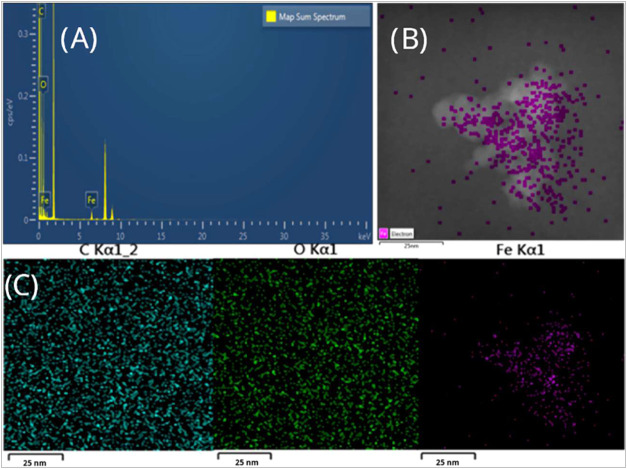
(a) Energy-dispersive X-ray spectroscopy (EDS) of the MMIP-MA system. (b) STEM image employed for the analysis. (c) Mapping of the distribution and relative proportion (intensity) of C, O, and Fe was performed.

Once the presence of the polymeric coating on the magnetite nanoparticles was demonstrated by the previous techniques, thermal analysis of the polymer on the nanoparticles was carried out. As the proposed system is designed for drug-release applications, it is crucial to characterize the stability of the polymer coating on the nanoparticles. Therefore, thermogravimetric analysis (TGA) and differential scanning calorimetry (DSC) studies were conducted, and their results are presented below.

Considering that each MMIP-MNIP pair was synthesized under the same conditions and with the same proportions of functional monomers and cross-linkers, it can be assumed that their thermal properties do not change because they involve the same polymer. Figure 8 shows the DSC thermograms of the systems formed by the four functional monomers evaluated. In all cases, an exothermic peak is observed between 314 and 366 °C, which is attributed to polymer crystallization. Followed by an endothermic peak attributed to the polymer decomposition process.^25^

**Figure 8:**
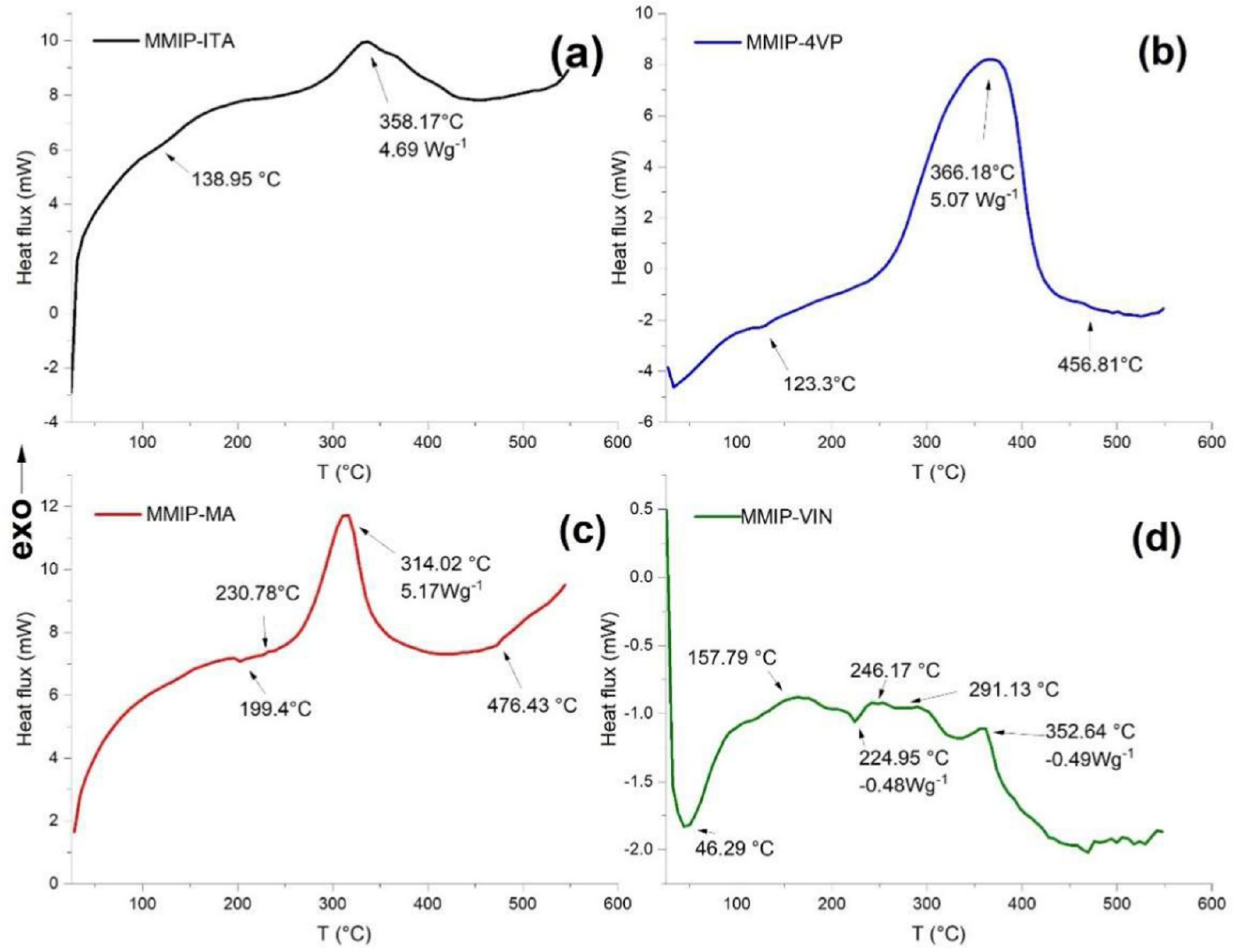
Thermal analysis by differential scanning calorimetry (DSC) of hybrid systems based on different functional monomers. (a) MMIP-ITA, (b) MMIP-4VP, (c) MMIP-MA and (d) MMIP-VIN.

TGA can be used to determine the relationship between the mass of a material and its temperature, as well as to evaluate its thermal stability and composition. To observe differences in weight loss between MNIPs and MMIPs, for imprinted samples, the analysis was performed without removing the drug from the polymer. The results obtained for all systems are presented in Figure 9.

**Figure 9:**
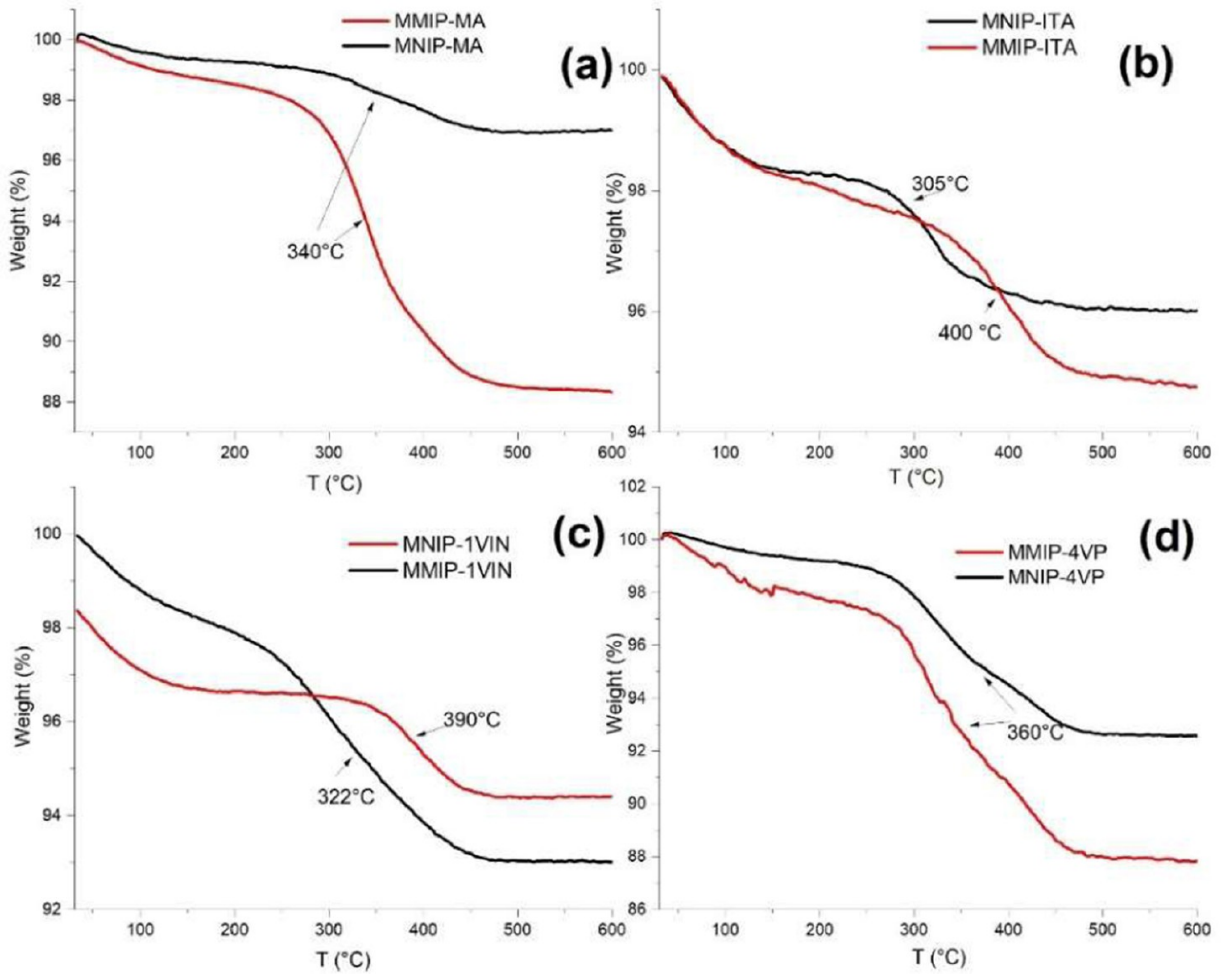
Results acquired from TGAs for the different systems. (a) MNIP-MA and MMIP-MA; (b) MNIP-ITA and MMIP-ITA; (c) MNIP-VIN and MMIP-VIN; (d) MNIP-4VP and MMIP-4VP.

For all systems, a weight loss of approximately 2% is observed between 100 and 120 °C, which is associated with the loss of solvent from the surface.^30^ After the initial drop in the curve occurs, a second weight loss is observed, which becomes more pronounced and is due to the decomposition of the organic material present in the sample. The MMIP systems lost more mass than the MNIP systems, which can be attributed to the presence of the drug in the polymer. MMIP-4VP and MMIP-MA showed higher weight loss compared to other MMIP systems, around 12% vs approximately 6% for MMIP-VIN and MMIP-ITA. This could be due to these systems being able to absorb greater amounts of drug, or because the polymer coating on these particles was more abundant. Finally, the decomposition temperatures of MMIP-VIN and MMIP-ITA are shifted relative to their respective NIPs, which can be attributed to the presence of the drug, causing greater stability in the polymer as it interacts better with the polymer network.

### 3.2 Synthesis and Characterization of Hybrid Magnetic Nanoparticles via Magnetic Hyperthermia

When superparamagnetic particles like magnetite are exposed to an alternating magnetic field, they exhibit the phenomenon of magnetic hyperthermia. This phenomenon can be used to initiate the polymerization reaction on the surface of nanoparticles, resulting in better-defined systems with surface coating.^20,31^ It is expected that particles will reach the temperature required to initiate the formation of free radicals through the response of the initiator (AIBN) in the reaction mixture during the synthesis of the polymeric coating. Thus, the NIP-HPT system was synthesized by exposing the reaction mixture to an alternating magnetic field of 107 kHz and 17.7 gauss, instead of heating at 65 °C and without the presence of 6-MP. The reaction was carried out for 24 h, during which an alternating magnetic field was applied. Subsequently, the particles were washed to remove the reaction residues, and their characterization was performed. The techniques used to characterize this system are the same as those described above. The results obtained by each of these techniques are summarized as follows. To verify whether the polymeric coating of the nanoparticles could be obtained using this new method, FTIR spectroscopy and elemental analysis were performed. The resulting spectra are shown in Figure 10.

**Figure 10:**
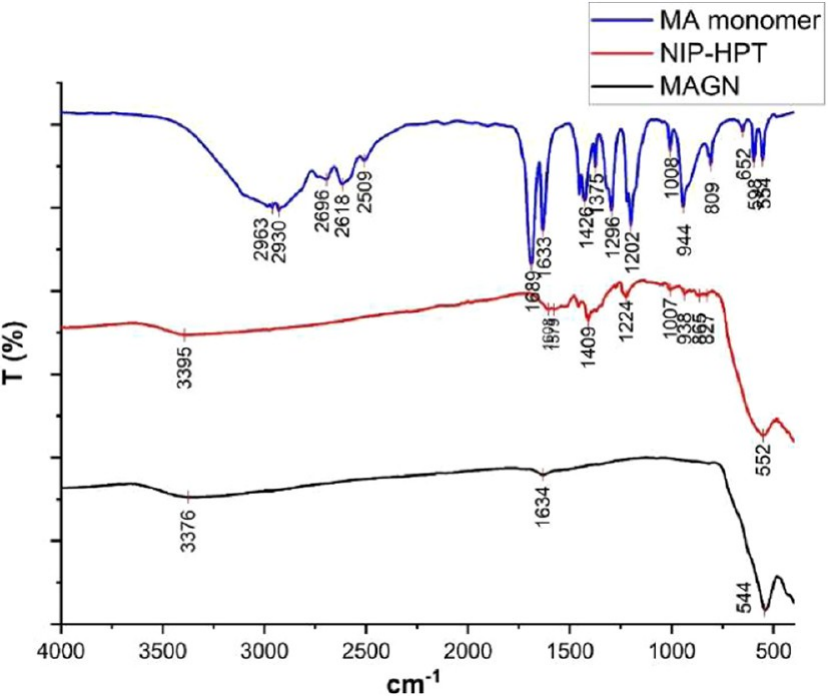
FTIR spectra of magnetite (black), functional monomer methacrylic acid (blue), and the NIP-MA-HPT system.

The presence of magnetite nanoparticles was confirmed by the signal at 552 cm^–1^, which is associated with the stretching of the Fe–O bond in magnetite. On the other hand, the absorption band at 3395 cm^–1^ in the NIP-HPT system is attributed to the presence of carboxylic acid groups, which is confirmed by the appearance of a band at 1608 cm^–1^. Additionally, other low-intensity and broad signals were observed, making it difficult to assign them to specific vibrations.

The elemental analysis of the NIP-HPT system yielded the following weight percentages: C, 2.8%; N, 0.24%; and H, 0.7%. This analysis confirmed the presence of the polymer on the nanoparticles, as the percentage of carbon (the main element in the polymer) was higher than the value found for single magnetite, which was 0.295%.

To verify that the crystal structure of magnetite did not undergo changes after exposure to an alternating magnetic field, X-ray powder diffraction analysis was performed on the NIP-HPT system. The obtained diffraction pattern (Figure 11) was analyzed using the MATCH!-Phase identification from the powder diffraction (PDR) ver. 3.5.0.99 software, showing 90% similarity to magnetite according to the JPDS number 00–019–0629.

**Figure 11:**
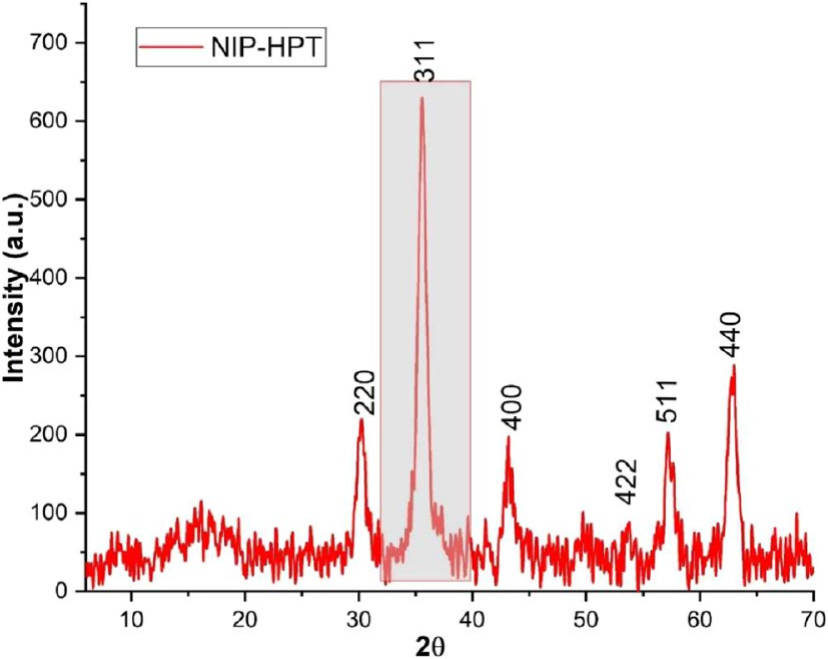
XRD patterns of the NIP-HPT system. The shaded region shows the maximum intensity peak for Gaussian fitting.

The diffraction peaks associated with the Miller indices (220), (311), (400), (422), (511), and (440) match with the characteristic planes of magnetite. Gaussian fitting was performed again on the peak of maximum intensity at 35.59° using OriginPro software, yielding a full width at half-maximum (FWHM) value of 0.89592. These data were then substituted into the Debye–Scherrer equation, resulting in an average crystal size of 9.73 nm.

The particle size and ζ-potential of the NIP-HPT system were determined at 3 and 24 h (NIP-HPT-3H and NIP-HPT-24H), for control particles at 1 and 24 h (CNT-1H and CNT-24H), and for particles functionalized with methacrylic acid (FUN-HPT) using DLS. The samples were resuspended in deionized water and sonicated for 10 min before measurement. The obtained results are presented in Table 3.

**Table 3:** Particle Size and ζ-Potential of the NIP-HPT System at Different Times

system	size (nm)	PDI	ζ-potential
FUN-HPT	640.1	0.566	–18.7
CNT-1H	474.7	0.47	–15.1
CNT-24H	651.7	0.504	–0.199
NIP-HPT-3H	648	0.598	–13.7
NIP-HPT-24H	669.3	0.504	–14.5

For all phases of the polymerization under hyperthermia and the control particles (without hyperthermia), particle sizes of approximately 600 nm were obtained. A slight increase in particle size was observed as the synthesis time increased (functionalized: 640 nm; after 3 h: 648 nm; and after 24 h: 669 nm). The obtained PDI values in all cases were close to 0.5 or higher, indicating the polydispersity of the analyzed samples. Finally, the ζ-potential of all samples was negative and never less than −30 mV, indicating that the particles were unstable in dispersion and tended to agglomerate.

For TEM analysis, samples were taken at different reaction times (*t* = 0, 1, and 24 h) while polymer synthesis was carried out by hyperthermia; for *t* = 0, it refers to magnetite particles functionalized with methacrylic acid under exposure to a magnetic field of 107 kHz and 17.7 gauss for 2 h. For samples at 1 and 24 h, portions of the reaction mixture were collected and washed with acetonitrile, and residues were separated magnetically. The obtained micrographs are shown in Figure 12. The polymeric coating in the functionalized particles [Figure 12a] exhibits a width lower than that in the particles polymerized by hyperthermia for 1 h [Figure 12c], fluctuating from approximately 4.5 to 7 nm. For this reason it is possible to confirm that the polymerization reaction was successfully induced by the magnetic hyperthermia effect on the nanoparticles. In the micrographs obtained after 1 h of reaction, high polymer yields were observed, which did not uniformly cover the agglomerated nanoparticles uniformly. At 24 h [Figure 12b], huge agglomerates of polymer coatings with a width of around 77 nm is observed and incrusted nanoparticles are noticed.

**Figure 12:**
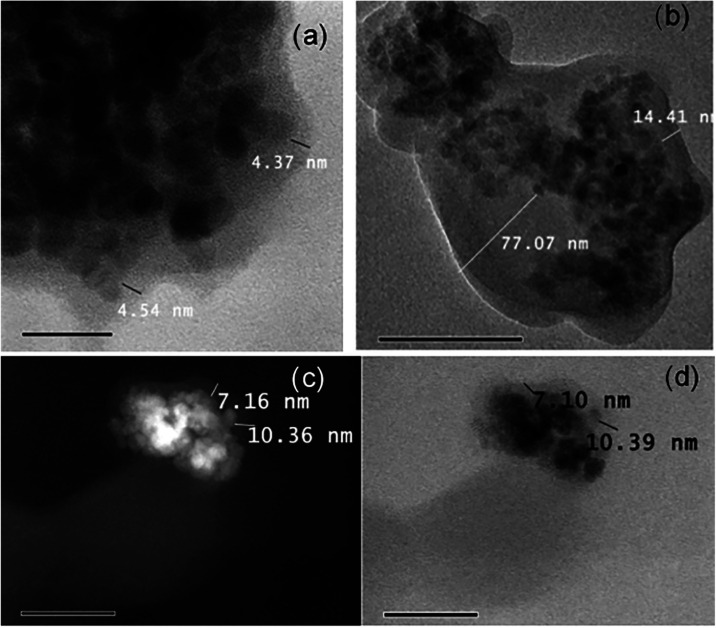
(a) Transmission electron microscopy image of NIP-HPT at *t* = 0. (b) TEM micrograph of the NIP-HPT in *t* = 24. (c) *Z*-contrast images of the NIP-HPT system after 1 h of reaction. (d) Image of the NIP-HPT system after 1 h of reaction.

The EDS analysis results of the NIP-HTP system are shown in Figure 13. In the insets (a) is evident the polymer presence coating in the nanoparticles and, in the insets (c–e), color regions display zones where C, O, and Fe atoms are present in the sample, respectively. The percentages of each element obtained by this method are C 50.35%, O 37.58%, and Fe 11.07%. These results are consistent with the high polymer coating thickness.

**Figure 13:**
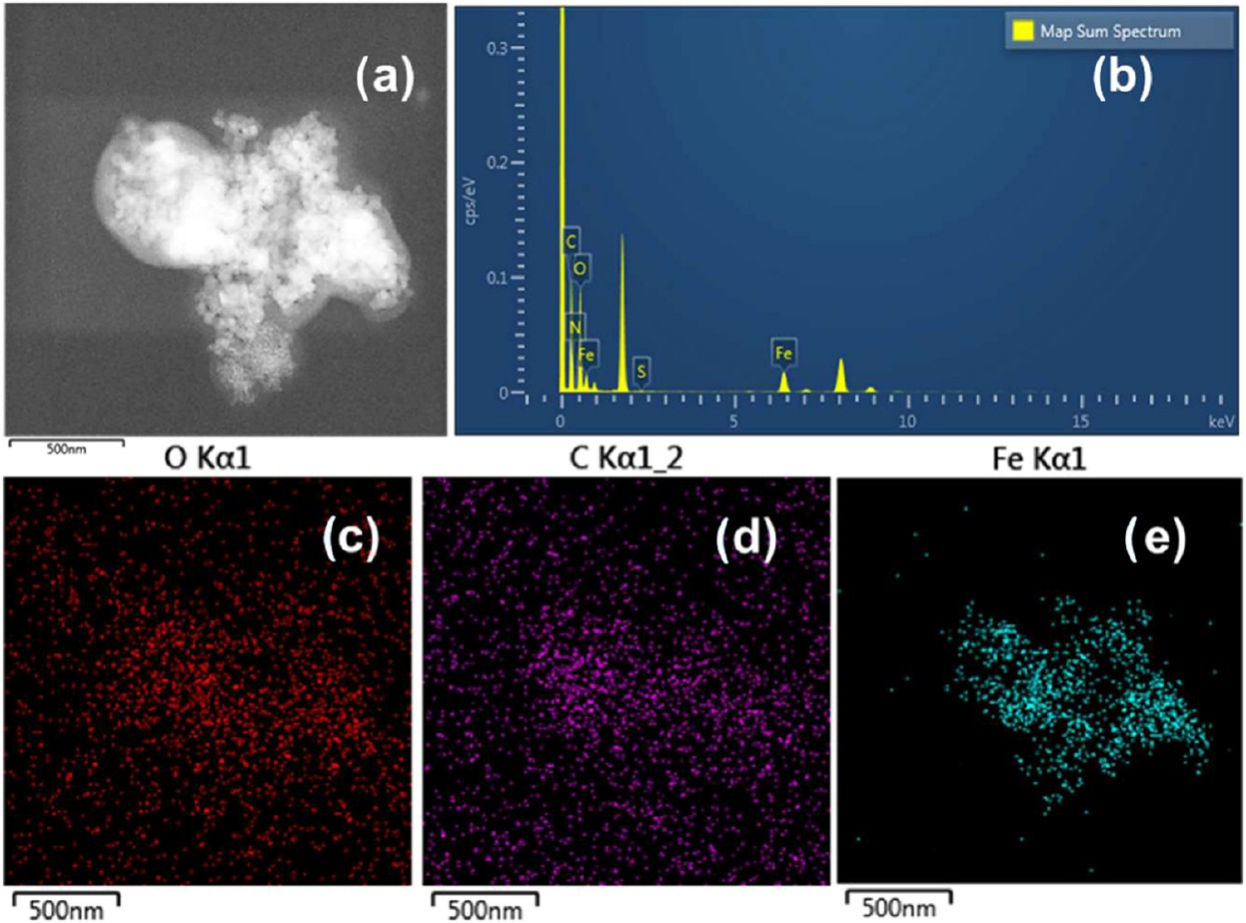
Samples were analyzed by EDS for the NIP-HPT system synthesized by hyperthermia after 24 h of reaction. (a) STEM micrograph employed for the analysis, (b) EDS analysis and (c–e) mapping of the distribution and relative proportion (intensity) of O, C, and Fe.

The magnetic properties of the synthesized nanomaterials were tested by Vibrating-sample magnetometry, hysteresis curves of each system were measured at 300 K and are summarized in Figure 14. As is noticeable, there is no observable hysteresis in any sample, which suggests that systems are superparamagnetic. Magnetization saturation values in nanomaterials were approximately 60 emu/g, whereas for magnetite particles without coating, this value was approximately 100 emu/g, indicating that saturation of magnetization decreased in coating systems, which is consistent with previous works published.^20^ Magnetization depends on the mass; for this reason, it is possible to affirm that if there is a 40% decrease in the magnetization value, this is the percentage (aprox.) Polymers present in the MMIP evaluated systems.

**Figure 14:**
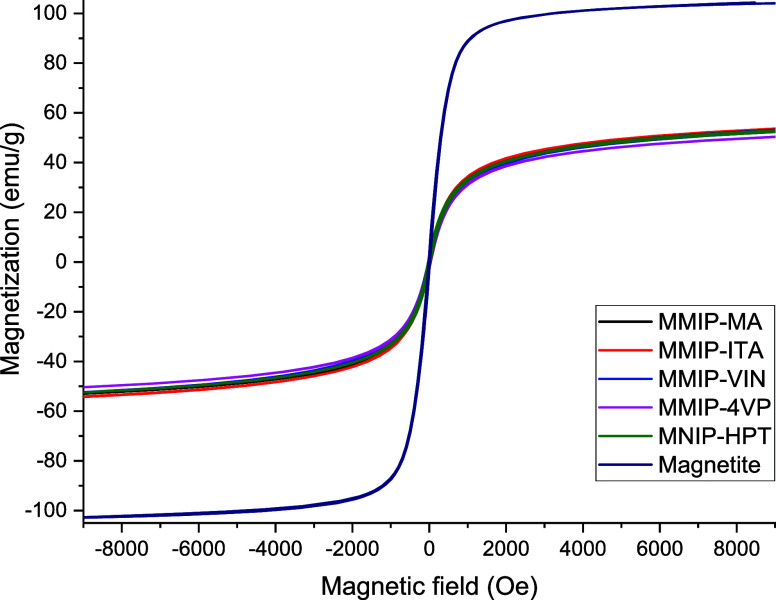
Hysteresis curves of materials prepared by magnetic hyperthermia.

### 3.3 Evaluation of Synthesized Drug Delivery Systems

Adsorption studies were conducted on the synthesized systems with and without the use of magnetic hyperthermia, as shown in Figure 15.

**Figure 15:**
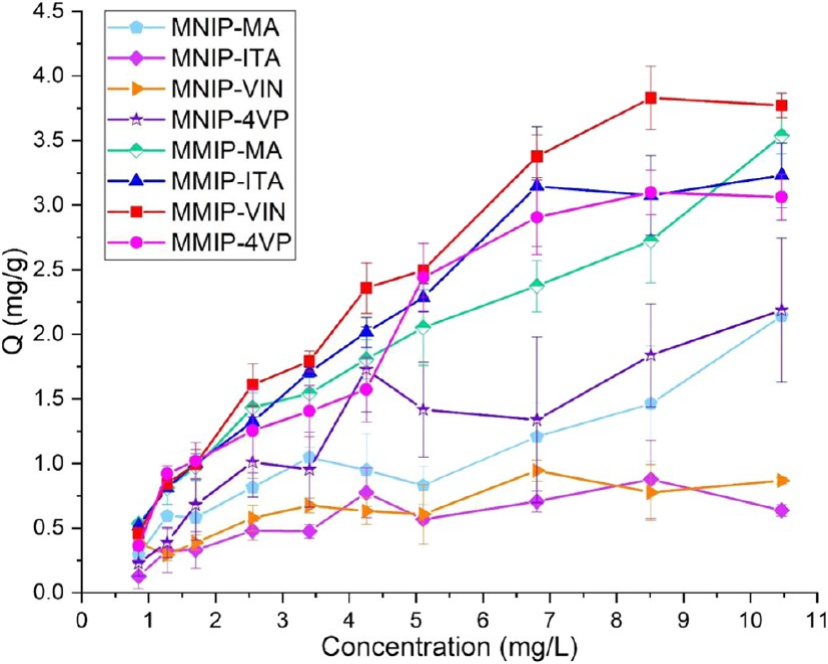
Adsorption isotherms for different MMIP and MNIP’s systems.

The isotherms were fitted into different models to determine the adsorption characteristics of the synthesized systems; each point on the isotherm was taken at 8 h while maintaining agitation (250 rpm) and temperature (25 °C) conditions. The obtained fits for each system are presented in Supporting Information (SI). The models used for the fittings were Langmuir, Freundlich, and Langmuir–Freundlich,^32^ as these are the models that best describe the adsorption mechanism on MIPs. Langmuir–Freundlich isotherm describes heterogeneous systems in which surfaces with binding sites exhibit different affinities.^33^ The best fit was expected because MIP systems often generate sites of varying affinity, depending on how the template molecule (6-MP in this case) interacts with the functional monomers during the prearrangement stage.

This is especially true for MIPs based on a noncovalent approach because different sites can potentially undergo potential interactions. Table SI1 lists the parameters for the tested models. For Langmuir–Freundlich isotherm, *N* represents the parameter related to the total number of high-affinity binding sites. When comparing systems with polymers of the same nature (MMIPs vs MNIPs) It was observed that systems with specific binding sites for 6-MP had larger *N* values than those synthesized without specific sites. This provides evidence of the correct formation of specific sites for 6-MP in MMIP systems. On the other hand, material heterogeneity is given by parameter *m*, and it can be observed that among the systems fitting the Langmuir–Freundlich model, only the MMIP-MA and MNIP-VIN systems are heterogeneous with *m* < 1. Heterogeneity may be due to the irregular coating of the polymer onto the particles. Finally, parameter a is related to the number of bioaffinity binding sites (*k*_0_) in the evaluated material. These bioaffinity sites can be calculated using the expression: *k*_0_ = *a* ^1/*m*^. Table 4 presents the values of *k*_0_ for the systems that fit the Langmuir–Freundlich model.^33^ The observed trend for most MMIP-MNIP pairs was that calculated *k*_0_ was higher in nonimprinted systems than in imprinted systems because the latter systems have a higher number of high-affinity binding sites. The imprinting factor indicates that MMIP-VIN possesses the highest affinity sites for recognizing 6-MP. In contrast, the affinity sites generated with VIN and 4-VP polymers exhibit lower imprinting factor values. It is important to note that the MNIP-MA material does not fit the Langmuir–Freundlich model; therefore, its data is not included in the table.

**Table 4:** Binding Midaffinity Sites and Imprinting Factor Calculated for Systems Adjusted to the Langmiur–Freundlich Isotherm

*k*_0_ = *a* ^1/*m*^	MMIP-MA	MMIP-ITA	MNIP-ITA	MMIP-VIN	MNIP-VIN	MMIP-4VP	MNIP-4VP
*k*_0_	5.95 × 10^–3^	0.15	0.69	0.35	0.02	0.47	0.50
imprinting factor		0.21		17.5		0.94	

#### 3.3.1 *In Vitro* Release Tests

For *in vitro* release tests of each system, solutions were prepared in phosphate buffer at pH 7.4, and the temperature was maintained at 37 °C to simulate physical conditions. Samples were collected at 0.25, 0.5, 1, 2, 3, 4, 5, 6, 7, 24, and 30 h. The concentration of 6-MP released was quantified by UV–vis spectroscopy. The obtained kinetics were fitted using different statistical models to determine the mechanism governing the release of 6-MP from the systems. The statistical parameters obtained from the model fittings are presented in SI.

Figure 16 shows the release profiles of all synthesized systems. It was observed that, despite washing with methanol before starting the release studies, all the systems exhibited a pronounced burst effect in the early measurement times, reaching up to 60% in some systems. However, a significant difference was observed between the amount of 6-MP released from the MMIP and MNIP systems. The last ones release all the adsorbed drugs in a shorter time due to the weaker interactions of 6-MP with the surface where no specific sites are present, leading to immediate release.

**Figure 16:**
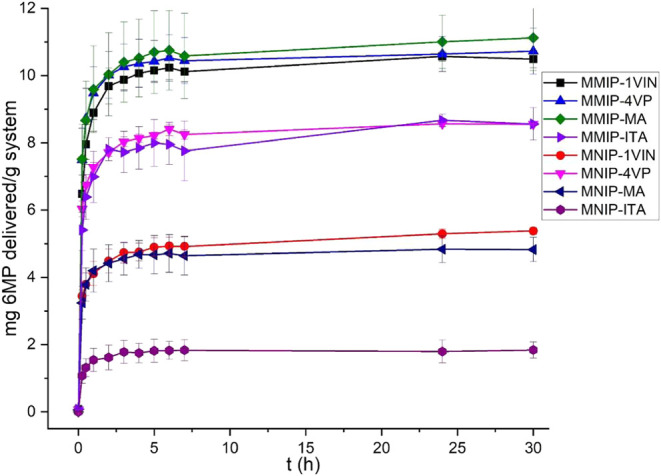
Release profiles of the synthesized systems.

When comparing the statistical parameters of the model fittings for each system, it was observed for all systems (except for the MNIP-VIN) that Peppas–Sahlin model provided the best fit to the presented release profiles. This was confirmed by the Peppas–Sahlin model, which obtained the highest model selection criterion, correlation coefficient, lowest sum of squares, and lowest Akaike criterion. This model suggests two mechanisms for drug delivery in the system: (1) the first mechanism for Fick diffusion, which is related to the *k*_1_ parameter, (2) and a second mechanism associated with an increase in the contact area in the system due to swelling in the polymeric matrix, which increases drug mobility through the media. The latter mechanism is correlated with the *k*_2_ parameter.^34^

As shown in Table 5, parameter *k*_1_ had the highest value, indicating that the drug release mechanism is exclusively governed by Fickian diffusion. This is further confirmed by analyzing the obtained values for parameter *k*_2_, where negative values are observed for all systems, indicating that the contribution of the release mechanism involving relaxation of polymeric chains is negligible or simply nonexistent. The values of parameter n corroborate the aforementioned findings, as all evaluated systems yielded *n* values <0.5, indicating a drug-release mechanism dominated by Fickian diffusion.^35^

**Table 5:** Calculated Parameter for Nonlinear Peppas–Sahlin Adjustment in the Different Systems

Peppas–Sahlin (*f_t_* = *k*_1_ *t^n^* + *k*_2_ *t* ^2*n*^)			
system	*k*_1_	*k*_2_	*n*
MMIP-MA	106.7	–27.5	0.1
MNIP-MA	97.8	–21.9	0.1
MMIP-1VIN	114.9	–32.5	0.1
MMIP-ITA	94.1	–22.5	0.1
MNIP-ITA	102.5	–30.5	0.1
MMIP-4VP	109.1	–29.6	0.1
MNIP-4VP	132.1	–43.3	0.1

#### 3.3.2 Magnetic Hyperthermia Release Tests

Release studies with MMIP systems were conducted in the presence of an alternating magnetic field to determine whether there was any change in the *in vitro* release profiles due to the hyperthermia effect of the magnetite nanoparticles under these conditions. MNIP materials are not included in this section, as it was previously demonstrated that they are not as effective as DDSs compared to MMIPs synthesized with the same monomer. Physiological conditions were also established for the tested samples and were measured at the same time intervals used for *in vitro* release (0.25, 0.5, 1, 2, 3, 4, 5, 6, 7, and 24 h). The amount of released 6-MP was quantified using the UV–vis spectroscopic method. The release profiles of the systems are shown in Figure 17.

**Figure 17:**
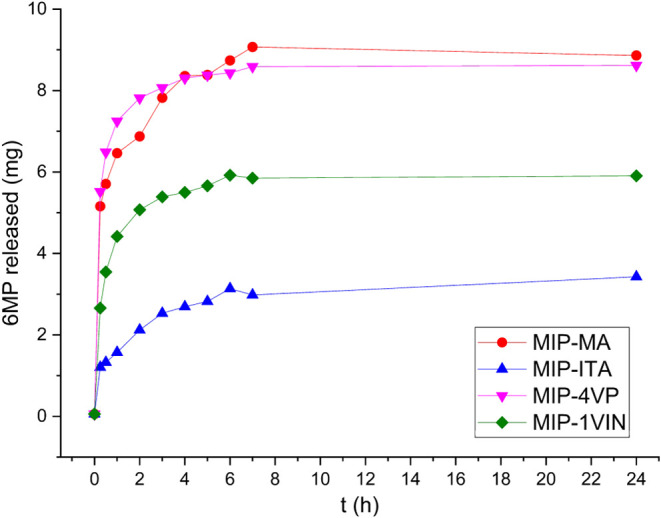
Release profiles of MMIP systems under an alternating magnetic field.

A reduced burst effect was observed in the MMIP-ITA system, and the amount of drug released when hyperthermia is applied is lower compared to when no alternating fields were applied. As shown in Table 6, the amount of 6-MP released for each system is lower when hyperthermia is applied, regardless of the time. Although both assays were conducted using the same batch of systems with adsorbed 6-MP, the amount of adsorbed 6-MP was the same for both conventional and hyperthermia-based release studies.

**Table 6:** Volume of Released 6-MP for MMIP Systems with and without the Use of Hyperthermia during Release

system	maximum amount delivered by hyperthermia (mg)	maximum amount delivered without hyperthermia (mg)
MMIP-MA	8.84	12.20
MMIP-ITA	3.25	9.48
MMIP-4VP	8.58	12.10
MMIP-1VIN	5.92	11.00

As shown in Figure 18, the amounts of released 6-MP differed for each system depending on the release conditions. It is important to mention that alternating magnetic fields can induce changes in highly cross-linked polymer structures, which increases the difficulty of drug delivery. This is supported by DSC MMIP and MNIP analyses (Figure 8), which reveal polymer crystallization when temperatures around 300 °C are reached. The resulting materials should undergo this kind of structural rearrangement due to their exposure to an alternating magnetic field, which is why small amounts of 6-MP are delivered. However, In the case of materials prepared with MA as a monomer, the difference in the release of 6-MP is not as significant as in the other cases (≈70%; 8.84 mg delivered with hyperthermia and 12.20 mg without hyperthermia) and the benefits that the rationalization of these magnetic materials provide for targeting the whole system to an affected region of the body by employing a noninvasive method as magnetism, the cost/benefit is justified. the MMIP synthesized using 4VP behaved similarly to MA-based MMIPs (Table 6).

**Figure 18:**
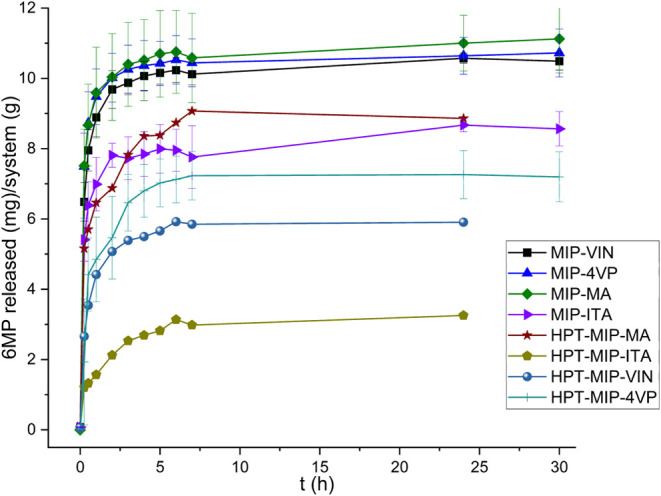
Comparative graph of the release profiles obtained for MMIP systems with and without the application of magnetic hyperthermia.

As observed in this work, the drug release induced by magnetic hyperthermia with molecularly imprinted polymers is moderate. It is clear that the behavior of the polymer as a function of temperature determines the release. Gracia-Mora et al.^36^ reported that magnetic nanoparticle systems coated with PNIPAMAm, which is a thermosensitive polymer, exhibit an enhanced performance. In this case, the authors report a typical LCST (Lower critical solution temperature) behavior, the polymer collapses when the critical temperature is exceeded, releasing 100% of the drug included in it. In our case, the effective cross-linking in the polymeric structure works as a suitable barrier for controlling the release. Another approach that demonstrates the importance of the characteristics of the polymeric matrix for release is the one made by Perera and co-workers,^37^ where they reported noncore–shell systems with poly­(vinyl alcohol) matrices and magnetic nanoparticles that induce triggered release. In this case, the polymer is not cross-linked, which makes the release faster when magnetic hyperthermia is induced. However, these systems may have rapid release kinetics by diffusion. In the proposed systems, the MMIPs are desing for an extended and controlled release through time.

## 4 Conclusions

Magnetite superparamagnetic particles were successfully synthesized by coprecipitation and organic–inorganic hybrid systems were prepared with molecularly imprinted polymers using different functional monomers, and the resultant materials were characterized accurately. An attractive method employing magnetic hyperthermia was used for the polymeric recovery of hybrid magnetic particles and the release of 6-MP because these hybrid materials were tested as DDS. Nonimprinted materials did not display as good performance as molecularly imprinted materials because the former lacks specific recognition sites as expected. Even all the systems tested displayed appropriate performances as DDSs, it was evident that the use of magnetic hyperthermia decreased the efficiency of drug release; however, for a pair of systems (MMIP-MA and MMIP-4VP), this reduction in the yield of 6-MP delivery was not substantial. This is important because the development of delivery systems able to carry drugs to target sites of the body using nonintrusive approaches like magnetism, is a relevant cutting-edge topic in science, and the final product needs to be evaluated in terms of cost/benefit analysis.

## Supplementary Material



## Notes

Adsorption isotherms obtained for MMIP′s and MNIP′s systems with the respective adjustments of Langmuir, Freundlich, and Langmuir–Freundlich models, associated coefficients to the employed models for isotherms fitting, and the statistic parameters for model adjustments for all DDS studied (PDF)
