# Supplementary material for: Organic–Inorganic Magnetic Nanoparticles Based on Magnetite Coated with Molecularly Imprinted Polymers for Drug Delivery Systems
Source: ACS Omega. 2025 May 19;10(21):21105–19. doi: 10.1021/acsomega.4c09515 (PMC12138690; doi:10.1021/acsomega.4c09515)
Supplement: Supplementary file 1 [file ao4c09515_si_001.pdf]

Organic-inorganic magnetic nanoparticles based of Magnetite coated with Molecularly Imprinted Polymers for Drug Delivery Systems

Sandra Ramírez-Rave[a], Leticia Antonio Gutiérrez [a], Iván D. Rojas-Montoya[a], M. Josefa Bernad-Bernad[b], Jesús Gracia-Mora[a*].

[a]Departamento de Química Inorgánica, Facultad de Química, UNAM, Avenida Universidad 3000, 04510, Ciudad de México, México.

[b]Departamento de Farmacia, Facultad de Química, UNAM, Avenida Universidad 3000, 04510, Ciudad de México, México.

* jgracia@unam.mx

Supplementary Information

Figure SI1. 6MP adsorption isotherms obtained for MMIP´s y MNIP´s systems with the respective adjustments of Langmuir, Freundlich and Langmuir-Freundlich models.

Table SI1. Associated coefficients to the employed models for isotherms fitting.

Table SI2. Statistic parameters for model adjustments in system NIP-VIN.

Table SI3. Statistic parameters for model adjustments in system MIP-VIN.

Table SI4. Statistic parameters for model adjustments in system MIP-MA.

Table SI5. Statistic parameters for model adjustments in system MIP-ITA.

Table SI6. Statistic parameters for model adjustments in system MIP-4VP.

Table SI7. Statistic parameters for model adjustments in system NIP-MA

Table SI8. Statistic parameters for model adjustments in system NIP-4VP

Table SI9. Statistic parameters for model adjustments in system NIP-ITA

Figure SI1. 6MP adsorption isotherms obtained for MMIP´s y MNIP´s systems with the respective adjustments of Langmuir, Freundlich and Langmuir-Freundlich models.

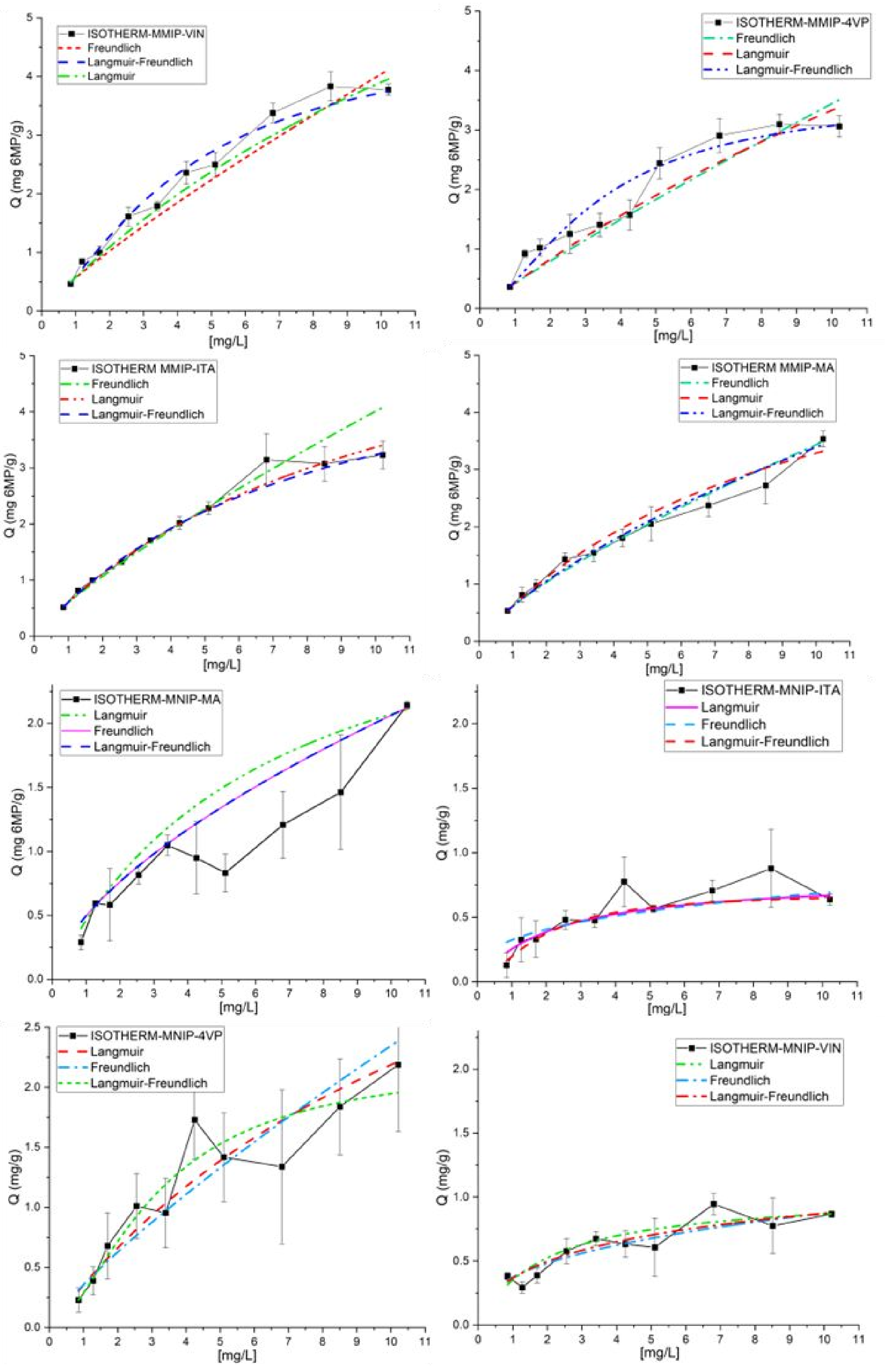

Table SI1. Associated coefficients to the employed models for isotherms fitting.

Coeficientes	MIP-MA	NIP-MA	MIP-ITA	NIP-ITA	MIP-VIN	NIP-VIN	MIP-4VP	NIP-4VP
Langmuir								
qs	6.43 ± 0.6	3.4±0.3	6.8± 0.6	0.81± 0.1	10.9± 2.8	1.03 ± 0.1	13.5±6.1	5.1 ±2.03
b	0.10 ±0.01	0.16 ± 0.02	0.1±0.01	0.45 ±0.1	0.05 ± 0.02	0.52 ± 0.11	0.03±0.01	0.074 ±0.04
Freundlich								
a	0.61 ± 0.01	0.5± 0.02	0.6 ± 0.01	0.32 ± 0.05	0.57 ± 0.03	0.37 ± 0.03	0.42 ± 0.01	0.36 ± 0.05
m	0.75 ± 0.02	0.62 ± 0.02	0.82 ± 0.02	0.33 ± 0.08	0.85 ± 0.04	0.37 ± 0.04	0.91 ± 0.04	0.82 ± 0.09
Langmuir-Freundlich								
N	18.9± 22.4	203.7±16674.7	5.6 ±1.2	0.7 ± 0.07	4.8 ± 0.9	1.7 ± 2.03	3.6 ± 0.7	2.2 ±0.4
a	0.01±0.03	6.31E-5 ±0.01	0.1±0.04	0.5 ± 0.1	0.24 ± 0.1	0.1 ± 0.5	0.3± 0.1	0.3±0.1
m	0.8±0.09	0.6 ±0.3	1.05±0.1	1.6 ± 0.5	1.4 ± 0.2	0.6 ± 0.4	1.6 ± 0.3	1.7 ± 0.3

Table SI2. Statistic parameters for model adjustments in system NIP-VIN

Parameters	Zero order	First order	Korsmeyer-Peppas	Peppas-Sahlin	Hopfenberg
Correlation coeficient (r^2)	-4.3941	0.7513	0.9912	0.6066	0.7512
MSC	-3.3409	-0.2109	2.9809	-0.8233	-0.3650
AIC	139.7644	100.3398	58.8458	108.3012	102.3426
SS	40025.1547	1928.6959	67.9594	3050.8201	1929.1154

Table SI3. Statistic parameters for model adjustments in system MIP-VIN

Parameters	Zero order	First order	Korsmeyer-Peppas	Peppas-Sahlin	Hopfenberg
Coeficiente de correlación (r^2)	-4.6448	0.9054	0.9643	0.9921	0.9054
MSC	-3.3647	0.7245	1.5320	2.7060	0.5572
AIC	130.8384	81.7681	72.0785	57.9898	83.7758
SS	46006.3479	770.7340	290.9676	64.4454	771.2258

Table SI4. Statistic parameters for model adjustments in system MIP-MA.

Parameters	Zero order	First order	Korsmeyer-Peppas	Peppas-Sahlin	Hopfenberg
Coeficiente de correlación (r^2)	-4.9826	0.7558	0.9798	0.9992	0.7557
MSC	-3.6213	-0.4228	1.9026	4.8403	-0.5896
AIC	129.8370	91.4549	63.5502	28.2973	93.4564
SS	42322.7530	1727.7563	142.9528	5.4273	1727.9590

Table SI5. Statistic parameters for model adjustments in system MIP-ITA

Parameters	Zero order	First order	Korsmeyer-Peppas	Peppas-Sahlin	Hopfenberg
Coeficiente de correlación (r^2)	-4.5026	0.4033	0.9779	0.9956	0.4030
MSC	-3.3728	-1.1513	1.9917	3.2897	-1.3057
AIC	138.0184	109.1379	68.2792	51.4049	111.1451
SS	34994.6765	3794.7681	140.4086	28.1864	3796.8772

Table SI6. Statistic parameters for model adjustments in system MIP-4VP

Parameters	Zero order	First order	Korsmeyer-Peppas	Peppas-Sahlin	Hopfenberg
Coeficiente de correlación (r^2)	-5.1549	0.7043	0.9761	0.9997	0.7043
MSC	-3.7376	-0.7018	1.6616	5.6479	-0.8559
AIC	140.7114	101.2467	70.5224	18.7002	103.2497
SS	43049.4487	2068.0486	166.8518	2.2775	2068.5245

Table SI7. Statistic parameters for model adjustments in system NIP-MA

Parameters	Zero order	First order	Korsmeyer-Peppas	Peppas-Sahlin	Hopfenberg
Coeficiente de correlación (r^2)	-4.9665	0.5928	0.9753	0.9937	0.5928
MSC	-3.5910	-0.9063	1.7306	2.7647	-1.0731
AIC	128.7574	96.5405	64.8975	52.4890	98.5419
SS	38681.4223	2639.5879	159.9389	40.7483	2639.8985

Table SI8. Statistic parameters for model adjustments in system NIP-4VP

Parameters	Zero order	First order	Korsmeyer-Peppas	Peppas-Sahlin	Hopfenberg
Coeficiente de correlación (r^2)	-3.1782	0.7026	0.3364	0.9996	-0.7544
MSC	-3.8442	-1.2015	-2.2042	4.9344	-2.9765
AIC	106.3206	79.8940	89.9208	18.5325	97.6416
SS	33930.1453	2414.8649	5388.8151	2.8670	14245.0507

Table SI9. Statistic parameters for model adjustments in system NIP-ITA

Parameters	Zero order	First order	Korsmeyer-Peppas	Peppas-Sahlin	Hopfenberg
Coeficiente de correlación (r^2)	-4.9595	0.5766	0.9420	0.9938	0.5765
MSC	-3.2451	-0.6007	1.2327	3.1585	-0.7547
AIC	139.8549	105.4773	81.6434	56.6077	107.4790
SS	40304.7212	2863.4823	392.5115	42.0582	2863.8619
